# Perceptions of the social determinants of health by two groups more and less affiliated with public health in Canada

**DOI:** 10.1186/1756-0500-6-247

**Published:** 2013-07-01

**Authors:** Lynn McIntyre, Robert Shyleyko, Cherie Nicholson, Hope Beanlands, Lindsay McLaren

**Affiliations:** 1 Department of Community Health Sciences, Faculty of Medicine, University of Calgary, Teaching Research & Wellness (TRW) Building, Room 3E14 (3rd Floor), 3280 Hospital Dr. NW, Calgary, Alberta T2N 4Z6, Canada; 2 University of South Australia, Adelaide, South Australia, Australia

**Keywords:** Social determinants of health, Equity, Health disparity, Health inequality, Canada

## Abstract

**Background:**

Despite strong academic recognition of the SDOH both in Canada and internationally, acknowledgement and uptake of the SDOH in health policy and public consciousness have remained weak. This paper aims to discern reasons for limited action on the SDOH by examining the perceptions of the SDOH held by two groups more and less affiliated with public health in Canada.

We conducted formal consultation with group members on their interpretation of the SDOH and their thoughts on the nature and basis of differences between those more and less aligned with the SDOH as a basis for action. Thematic analysis was used to evaluate the views of the two groups.

**Findings:**

Group 1 (community/public health workers) felt overwhelmed when confronted with questions regarding action on the SDOH within the context of their professional lives. They suggested an expanded list of health determinants that included factors such as voluntarism and happiness, transcending traditional notions of “root causes.” Furthermore, they did not articulate value-based reasons why others would oppose the SDOH; rather, in line with their professional roles, they adopted a value-neutral and pragmatic approach to working to improve health. Group 2 (child and youth advocacy organization members) seemed rooted in the 1986 Ottawa Charter for Health Promotion framework, with their recommendations aligned with strategies such as building healthy public policy and reorienting health services. Neither group made reference to issues of social justice or inequity when they made suggestions for improving health.

**Conclusions:**

We found that two groups with different affiliations to formal public health could discuss the SDOH without acknowledging the inequitable distribution of power and resources that lies at its root. We also found that those working in public health had difficulty moving beyond individual actions that they or their clients could take to improve health. For a group more focused on advocacy than direct service provision, the Ottawa Charter framework seemed more easily suited to their recommendations for action than suggesting actions that would address the SDOH. Our findings indicate that there remains work to be done in terms of translating the SDOH concept into action in Canada.

## Background

The social determinants of health (SDOH) are rooted in the recognition that the determinants of illness and disease transcend the individual. The focus of the SDOH is on the contexts in which individuals and communities “are born, grow, live, work and age” [1], p.1]. The academic literature is replete with strong and consistent evidence for the relationship between multiple socioeconomic factors and differential health outcomes [1-6].

Research into and acknowledgement of the SDOH culminated in 2008, when the World Health Organization (WHO) released its final report from the Commission on the Social Determinants of Health (CSDH) [1]. The CSDH has been instrumental in establishing health equity, as a global concern requiring “urgent and sustained action” from local to international levels of governance [1], p.1]. The 2008 CSDH report synthesizes international evidence on the SDOH, highlights contemporary inequalities in health, and provides recommendations to reduce these inequalities. The report implicates social inequity as the principal driver of differential health outcomes, thereby departing from the list of health determinants often cited in other jurisdictional reports [7-9]. Furthermore, recommendations are made to address avoidable health inequalities by targeting “the inequitable distribution of power, money, and resources – the structural drivers of those conditions of daily life – globally, nationally, and locally” [1], p.2].

Canada has been an active participant and supporter of the CSDH; most notably, Canada provided funding assistance to three of the CSDH’s nine knowledge networks, conducted a global review of intersectoral action for equity in health [10], and was represented on the CSDH by a Commissioner, the Honourable Monique Bégin. Canadian public policy attention to the CSDH has also been apparent. 2005 saw the establishment of the federally-funded National Collaborating Centre for Determinants of Health whose mandate is to build public health capacity to address the SDOH. In 2008, the Standing Senate Committee on Social Affairs, Science and Technology, Senate Subcommittee on Population Health was given and fulfilled a mandate to “examine and report on the impact of the multiple factors and conditions that contribute to the health of Canada’s population – referred to collectively as the determinants of health” [11], p.vii]. The Chief public health Officer’s (CPHO) 2008 *Report on the State of public health in Canada*[12] and the Conference Board of Canada’s 2008 report on the *Case for Business Action on the Socio-Economic Determinants of Health*[13] further represent the policy attention given to the SDOH in Canada.

### Limited action on the SDOH and possible reasons

Despite this apparent policy interest, tangible action on the SDOH within health policy in Canada has been limited [14-18]. Most notably, the SDOH were absent from the Canadian federal election campaign which occurred concurrently with the CSDH Report’s 2008 release, and from all party health care platforms. Several spheres of influence that may account for the limited uptake of the SDOH in Canada have been identified. Among these are weak public support, lack of understanding and prioritization by health and community-level decision makers, inadequate media attention, sociopolitical challenges with using the SDOH as a basis for action, and limited scope of public health practice. These are highlighted below.

Public opinion remains rigid on attributing health disparities to individual health choices and not social determinants [16,19,20]. Reutter and colleagues have noted that “public opinion plays an important role in legislated social policy” [21], p.13], and thus, the public’s perception of the relationship between health and social determinants can directly influence the degree and direction of support they voice for social health policies and programs [21]. Indeed, Canadian public opinion has been split for some time in term of readiness to take action on the SDOH. In 1996, Reutter et al. conducted a survey of over 1200 Albertans and found that 41% of the lay public perceived structural factors (specifically, living circumstances) as the best explanation for the relationship between health and poverty, while 20% perceived behavioural factors as the best explanation [21]. A 2008 analysis of public responses to media coverage about food insecurity as an exemplar of health inequality showed both agreement with the determinants of health message and strong disagreement, in the form of negative, personal responsibility perspectives [22].

In 2000, Lavis and colleagues surveyed 153 Canadian civil servants occupying decision-making posts in social services, labor and finance departments, both provincially and federally, and found a lack of understanding about the SDOH and prioritization among these health and community-level decision makers. 50% of respondents were familiar with the SDOH, and 65% felt that knowledge about these ideas had had some policy influence in their sectors; 83% however, expressed the need for information on effective intervention strategies. Respondents from the finance sector were consistent outliers with respect to both familiarity and attitude with/towards the SDOH framework and did not see the ideas about the determinants of health as relevant for major government initiatives [23]. Two research groups conducted analyses on the perception on the SDOH by key stakeholders in provincial health (Prince Edward Island and Saskatchewan) in Canada in the late 1990s [19,24]. Health system employees as well as key decision makers were asked to assign priority rankings to the determinants of health they felt were most influential on the health of their community. “Personal health practices” were ranked as most influential on health by the respondents in the PEI study [19]. Kahan and Goodstadt similarly found that, although the majority of respondents agreed that the health of a population is strongly affected by social justice, only few thought that high priority should be given to shifting resources towards addressing equity issues [24]. In general, their survey results revealed significant inconsistencies in how respondents understood the determinants of health. Collins analyzed the perceptions of the SDOH held by “local actors” (individuals working for a community-based organization in Hamilton, Ontario, or the municipal government in Vancouver, British Columbia) [25]. Consistent with previous results, a high rating (for both, influence on health and priority for resources) was given to lifestyle-related determinants (“healthy lifestyle”), and much lower ratings to “income” or “social support” [25].

Media promotion of the SDOH framework has also been limited. For the time period 1993 through 2001, Hayes and colleagues found that only about 6% of health-related stories of Canadian newspaper media focused on the SDOH [26]. In 2002/2003, Gasher et al. interviewed Canadian health reporters on how they chose their health stories and found that, despite access to scholarly papers on the SDOH, they underreported the SDOH in favor of the importance of the health care system and personal health habits in health outcomes [27]. Recently though, André Picard, a public health reporter for a prominent Canadian newspaper, has challenged deeply rooted lay beliefs about health and illness in Canada [28], and has also given vital media attention to the findings of the CSDH and the SDOH [29].

Several authors have commented that a shift in Canada’s political and economic system towards a conservative neoliberal ideology and increased market orientation reduces opportunities to act on both inequities and determinants such as income [16,17,30,31].

Importantly, another potential reason for inaction could be that those whose jobs might be to act on the social determinants of health — public health practitioners for example, have been ill-equipped to do so. Raphael noted that public health practice in Ontario public health units focused on downstream determinants [20]. The National Collaborating Centre for Determinants of Health reported a similar finding for Canadian public health in general: in an environmental scan spanning the previous five years, they reported that public health action on broader health determinants was not widespread and suggested that it may even be viewed as “new” [9]. The report hypothesized that one reason for lack of public health uptake may have been that foundational concepts were never universally institutionalized, i.e., made a part of public health best practices [9]. Several authors have postulated other reasons why public health practice has been slow to adopt a SDOH approach citing inadequate knowledge transfer/translation between public health researchers and public health decision makers [30,32-35] and a dominant ideology of health promotion remaining behaviourally based [30]. Notwithstanding the numerous commentaries and critiques, empirical evidence derived from health professionals working in public health, or with public health, has been limited to date in Canada. In the light of this gap, it seems worthwhile to try to understand how individuals affiliated with public health to a greater or lesser degree understand the SDOH and their policy and program implications. Thus, this study aims to discern reasons for limited action on SDOH by examining the perceptions on the SDOH held by two groups more and less affiliated with Canadian public health.

### Consultation processes

Two groups with differing affiliations with Canadian public health were approached to discuss the SDOH (Table 1). Overall, the goals of the consultations were to understand how each group recognizes and reacts to the SDOH and to identify how each group interprets the barriers realized in communicating and taking action to address the SDOH. Group discussion and plenary feedback data from both groups were collected and analyzed using qualitative description, a design which is characterized as “an eclectic but reasonable combination of sampling, and data collection, analysis, and re-presentation” [36], p.334]. Ethical approval was received from the University of Calgary Conjoint Health Research Ethics Board.

**Table 1:** Group demographics and details related to group consultations

	**Group 1**	**Group 2**
**Event**	Summer School Plenary workshop: “Closing the Social Determinants of Health Paradigm Gap in Less than a Generation”	National non-governmental organization consultation on the social determinants of health
**Date**	August 2009	November 2009
**Number of Participants**	50 by head count estimate at start of session	12
**Participant Details**	Front-line public health and other health practitioners, staff and volunteers from community and non-governmental organizations (NGOs), multisectoral health managers, students and academic researchers, the latter two groups representing three universities. A majority of the registrants were female and were working within the government health sector at various levels.	Members of the Board of Directors and Advisory Council. Well-educated health, education, social service, and public administration professionals in their mid- to late careers, including a number of retired volunteers. Participants consisted of 10 women and 2 men.
**Pre-reading**	None	Provided; pre-reading material noted in *Methods*
**Questions Posed**	1. How would you describe to a layperson what the SDOH framework is, and what types of actions might be taken on the social determinants of health to reduce health inequalities?	1. What do you understand the SDOH framework to mean?
	2. Imagine a thoughtful person who rejects the SDOH notion – what would they say to those immersed in the SDOH paradigm, and what would they be most likely to argue against?	2. Do you see the SDOH framework as having merit and why? If not, why not?
	3. Identify the cleavage issues (philosophical, value-based) as well as the information deficits between the two views.	3. For the components of the framework that you see as having merit, how might we move things forward?
**Format of Discussion**	Participants were free to divide themselves among 8 groups, each group with its own specific topic focus to provide context for the discussion: tobacco, mental health, Aboriginal health, interpersonal violence, homelessness, food insecurity, and two open discussion groups.	Fishbowl technique for group discussion: 6 members involved in discussion sitting in an inner circle, while the remaining 6 members observed sitting in an outer circle. Switched after 45 min of discussion.
	Plenary feedback followed.	Plenary feedback followed.
**Duration**	2 hours of group discussion	45 minutes of group discussion for each group (total 90 minutes)
	30 minutes plenary feedback	30 minutes plenary feedback
**Recording Procedure**	Hand-written facilitator notes were taken throughout the discussion, and key points were summarized and shared in the plenary session. The emphasis of these reports were on ‘ah ha’ thoughts, i.e., moments of enlightenment, and ‘stuck’, unresolved issues.	All of the discussions were recorded with two digital recorders; handwritten notes by the project coordinator; facilitator flip chart notes.
**Consent**	Research ethics board approval. Multiple pre-notifications about the session were circulated to registrants and assent was obtained from all participants at the beginning of the workshop.	Research ethics board approval. Verbal consent was obtained from all participants at the beginning of the session.

### Group 1

(Community health workers, including formal public health workers and those working in community health-related organizations).

For our first consultation, we used purposive sampling to select a group that was expected to have a strong alignment with public health and hence reasonable knowledge of the SDOH and its program and policy implications [37]. We approached a provincial public health association that was hosting a three-day “Summer School” in August 2009, with an objective to examine the gap between knowledge and action respecting the many environments which contribute to health (including the social determinants of health): organizers agreed to our participation in the program. The stated goal of the Summer School was to increase the capacity of public health professionals to protect and promote health and prevent disease. Based on their interest in the SDOH and their positions in public health or community-based health services, as well as a curriculum focused on SDOH concepts, we expected that attendees would be more strongly affiliated with the SDOH relative to our other group. The program was targeted to those working to improve health and reduce health inequities in the public health sector, in the municipal sector, in the non-profit sector, and in other sectors that share this objective. The Summer School specifically sought to improve the skills and ability of public health sector staff and others working to improve health and reduce health inequalities.

Our session was scheduled for the final day of the Summer School, after attendees had received the full workshop curriculum, which included the equivalent of full sessions on “Introduction to Determinants of Health” and “Evidence Informed Decision Making in Public Health.” As Summer School attendees, Group 1 participants were broadly representative of individuals who work to improve health and reduce health inequalities in the public health, municipal, and non-profit sector. Following group assent (no attendee declined), all 50 or so attendees (the exact number of Summer School participants fluctuated day to day) self-selected into small groups in order to apply the SDOH either to a specific topic (tobacco, homelessness, food insecurity, aboriginal health, mental health, interpersonal violence) or more generally (two open groups). Group facilitators, who were faculty members or graduate students at the local university, recorded discussion notes from their small groups. The group discussion questions and further details of the methods used to conduct interviews and plenary discussions for this group can be found in Table 1. The recorded discussion notes comprised the data for analysis. Thematic analysis of discussion and plenary feedback data was conducted by classifying participant responses into identifiable themes to provide insight regarding reactions to and views on the SDOH [38].

At the time of facilitator debrief, we recognized an important limitation of the methods used with this group as well as a finding in itself. Our assumption that differing worldviews on the SDOH might be paradigmatic was challenged by our finding that asking participants to specifically consider whether or not differing views of the SDOH could be paradigmatic made an assumption that this group would perceive the SDOH with more uniformity of worldview than they did. Given that this assumption was incorrect, we modified our questions in our consultation with the second group.

### Group 2

(Child and youth advocacy organization members)

We again used purposive sampling to identify a group whose work was related to Canadian public health, but who did not work within a formal public health- or community health-related structure. We also sought a group that moved beyond the provincial level. These desired characteristics were met by the Board of Directors and Advisory Council of a national non-governmental organization dedicated to the health of children and youth. All members were approached, and 12 out of the 24 members travelled from various parts of Canada to participate in a half-day consultation. It should be noted that the first author is a member of the Advisory Council, but did not participate in the group discussion. Group participants were characterized as citizens with a demonstrated commitment to health, civic engagement, and social justice regarding children and youth issues. Because the organization does not incorporate the SDOH in its daily work, we expected this group to have a weaker affiliation with the SDOH than Group 1.

The questions posed to Group 2 participants, which structured discussion, are listed in Table 1, along with other pertinent details related to this consultation. When preparing for the consultation, it was suggested by staff and the Chair of the organization that members might wish to have access to materials that would aid in their discussion of the SDOH. This seemed to be a fair request given the first group had had an opportunity to reflect on information related to the SDOH in their Summer School curriculum. We prepared a list of optional pre-reading material, including the executive summary of the 2008 CSDH Report [1] and three other reports deemed important Canadian responses to the CSDH [11-13], as well as the Public Health Agency of Canada’s list of 12 key health determinants [10]. While we did not ask if participants had read any of the materials, as the discussion unfolded it appeared that most had at least linked to some of the documents for a cursory view. The methods for conducting discussions addressed limitations identified from the consultation with Group 1, most notably by the use of the fish bowl technique for facilitated discussion [39], digital audio-recording of discussions, and extensive note-taking. We also modified the wording of questions to be more neutral (i.e., no mention of paradigms) when asking about the SDOH.

Thematic analysis, based on the discourse captured from the detailed notes, was used to interpret, organize, and mediate understanding of the data. Here, a discourse is taken to mean a “belief, practice, or knowledge that constructs reality and provides a shared way of understanding the world” [40], p.24]. All discussion and plenary feedback comments by the participants fit into one of four emergent discourses that constituted the final themes for the group. Due to the fact that the non-governmental organization was focused on child and youth health, the issues and topics discussed related to the SDOH in the context of children, youth, and families.

## Findings

### Group 1

Following thematic analysis of the non-verbatim notes taken of the discussion, five overall themes were identified:

1) *Reconciling individual- versus population-level health.*

There appeared to be difficulty in applying the SDOH at the level of the population. Much of the discussions centered on individual cases and solutions regarding the SDOH.

2) *More determines health than is captured by a list of determinants or a notion of “root causes.”*

The group was particularly struck by earlier discussions in the Summer School that focused on love and happiness, as well as their own discussions on spirituality, aboriginal concepts of health, and voluntarism, which were argued to determine health just as much as Health Canada’s list of 12 determinants, i.e., income and social status; employment; education; social environments; physical environments; healthy child development; personal health practices and coping skills; health services; social support networks; biology and genetic endowment; gender; and culture [10].

3) *Action on the SDOH is overwhelming.*

If one accepts the CSDH’s recommendation to “tackle the inequitable distribution of power, money, and resources” [1, p.2], participants felt overwhelmed with respect to what they could do in their professional roles to act on the SDOH.

4) *Differences in views regarding the SDOH are not necessarily paradigmatic.*

Although participants agreed that there are distinct ways of understanding and characterizing the SDOH, the consensus was that shades of grey rather than polarized paradigmatic views characterized these differences. They were reluctant to “demonize” those who might oppose the SDOH framework, as they felt that this type of approach made understanding and action on disparities more divisive than it needed to be.

5) *Short-term versus long-term time horizon.*

The basic conceptualization of the SDOH was presented as a structurally-rooted set of conditions that led to health disparities. The time horizon for action and investment would therefore seem to be long-term, offering little to those who are faced daily with populations living with disparity, and who want to see results that would come from short-term solutions.

In general, the Summer School participants agreed that the SDOH are more than a(n) (incomplete) list of root causes and would be best conveyed to the general public through the use of simple language and stories/anecdotes that provide a “face” to disparity. They identified many reasons why SDOH frameworks might be rejected, including the possibility that acting on the SDOH could lead to a disruption of the social order. Participants were most comfortable with community-level, i.e., local action, although they also called for multi-sectoral action, i.e., reducing the silos, and for leadership at all levels of government. Overall, discussion took a pragmatic approach to the SDOH and improving health, rather than an examination of fundamental difference in worldviews that might influence perceptions or action on the SDOH.

### Group 2

Four themes captured the rich discussions from the fishbowl and follow-up plenary sessions; of note, participants usually spoke to more than one of these themes:

1) *Enlightened educational strategies are needed to convey the message regarding the SDOH*.

Participants highlighted the need for clear communication to support the SDOH while also acknowledging the advocacy challenge to acting on the SDOH. To this end, participants believed there was a need to inform public opinion in order to pressure government into action. This might be achieved through appropriate language and technologies to support a variety of information sharing, research dissemination, and instructional approaches to mobilize individual action and achieve a broader and deeper understanding of health and what determines health.

2) *The health care system can be used to augment the germaneness of the SDOH in the care of children and youth.*

Participants recognized the SDOH framework as a more in-depth understanding of health and representative of a deeper view into issues that lie behind lifestyle choices. There was also discussion on bringing the SDOH to the attention of all health professionals so they could provide leadership and act as advocates for changes to government policy. Participants called for primary health care integration, delivered by enlightened physicians who used evidence to show leadership in the improvement of child and youth services that attend to non-medical factors.

3) *Political engagement on the SDOH requires the participation of civil society to influence public opinion and ultimately political leaders*.

Participants expressed the need to measure and then mobilize community support that is consistent with the SDOH framework. They discussed governance, the role of civil society, processes to mobilize public opinion, and the implication of societal rules. They called for youth engagement, community activism, and an opportunistic dialogue with leaders.

4) *The fundamental premises of the SDOH may be flawed*.

Participants provided a critique of the sociopolitical system, including values and ideologies, associated with the SDOH. They asserted that the distinction given to the *social* determinants of health is irrelevant because all determinants, including human biology and the health care system, they argued, are socially constructed.

### Implications

Our objective was to discern reasons for the limited action on the SDOH by means of an analysis of the perspectives of two groups with differing affiliations to Canadian public health. By considering the findings from these groups we can understand possible reasons for the inadequate uptake of the SDOH, particularly among those charged with taking such action directly and those who work with them.

Group 1’s discussion of their difficulty in taking action on the SDOH in their professional lives is in line with the National Collaborating Centre for Determinants of Health’s analysis of public health capacity: the foundational concepts of the determinants of health have yet to be institutionalized, leaving implementation at an early adopter stage [9]. Their report suggests that challenges to more widespread action include the lack of clarity regarding what public health should or could do within a limited evidence base and preoccupation with behavior and lifestyle approaches – barriers we captured from members of the group as well. A preoccupation with behavior and lifestyle approaches among Canadian health workers, albeit not explicitly public health workers, has been reported before in another provincial jurisdictions [19,24,25]; thus, the findings may not be particular to Alberta, where there is entrenched conservatism (as illustrated by more than forty years of the same governing conservative political party). If anything, the Summer School’s focus on more abstract ideas about dealing with inequities and nuances around community action strategies such as voluntarism, coupled with the importance of spirituality and relationships between and among individuals, may have confused attendees regarding action on the social determinants of health to improve population health and reduce health inequities. In addition, a strong presence of aboriginal worldviews may have left the group with too many conceptual threads, leading them to retreat to their traditional public health practice methods and ideas, that are more clinical and individual than public policy directed. While not a failure of the SDOH *per se*, the SDOH did not serve as a conceptual framework for these community health workers to raise questions about what they could do to improve population health and reduce health inequities in their public health practice.

The themes arising from Group 2 conveyed many features of the 1986 Ottawa Charter for Health Promotion [41] and several key health promotion attributes, in particular public education, engaging health services, and calling for government involvement [18,41]. This might partly be explained by the fact that the establishment of their organization and its policy stances preceded the clear development of the SDOH framework. Of note, the Ottawa Charter was not among the readings provided to participants. Also of note, any pre-reading that the attendees might have done did not surface in a textured understanding of the SDOH.

While discussions of this group were more public policy-oriented than Group 1, there lay in their recommendations a fundamental belief that education of the public, politicians, media, and policy makers was needed in order for the SDOH to be better understood and appreciated. Indeed, it is not uncommon for authors who examine perceptions of various Canadian groups on the SDOH to include calls for education of these groups [14,17,24,42]. The landmark document by the CSDH also recommends that educational institutions “increase understanding of the social determinants of health among non-medical professionals and the general public” [1], p.189]. We suggest however that it is somewhat disingenuous to decry public health workers and others for defaulting to individual educational strategies in the name of advancing action on the SDOH when these very strategies are touted as viable mechanisms for changing the behaviours of policy makers.

Although the findings from the two groups arose from different methodologies and therefore cannot be directly compared, one observation is that both groups failed to reference inequity, i.e., inequitable distribution of power and resources [1], as a structural driver of differential health outcomes. A basis in health promotion should not have precluded this: the Ottawa Charter and contemporary Canadian health promotion discourses, including Epp’s *Achieving health for all: A framework for health promotion*[43] and Hamilton and Bhatti’s population health promotion framework [44], clearly identify inequity as a key structural determinant of health. Targeting inequity is asserted to be fundamental to population health promotion [44], as inequity is interwoven with larger social structures and thus affects health at the population level [18]. Group 2 diverged from the health promotion framework in that discussion focused on strategies aligning with other aspects of health promotion such as health professional education, youth engagement via social media, and primary health care integration. Group 1 also ignored inequity as the root cause of differential health outcomes. In fact, this group suggested that determinants of health transcend root causes of health inequalities, and that the SDOH is better described by multi-factorial causes and multi-pronged solutions that include factors such as happiness, love, spirituality, and voluntarism.

The CSDH’s “*holistic* view of social determinants of health” [1], p.1, emphasis added] focuses on “deep inequities in the distribution of power and economic arrangements, globally, which are of key relevance to health equity” [1], p.1]. The conceptualization of health disparities as arising from amongst a list of determinants permits a laundry list of strategies to be proposed (e.g., educate parents, get more people volunteering in their communities, break down the silos in health) that are disarticulated from structural inequity. It has been hypothesized that in Canada too much emphasis has been put on the SDOH, without considering that it is their unequal distribution that causes health inequities [14,16,45]. This might be a direct consequence of a “positivist” health science, reluctant to make normative judgments [14,46]. Alternatively, the policy implications derived from such judgments might conflict with existing ideologies/paradigms in current Western societies, where the concept of individualism seems predominant [14]. These more structuralist analyses reinforce the weakness of an educative, narrow focus on specific SDOH; however, structural critiques by their nature often do not lead to precise actions to remedy the problems they dissect.

If action on the SDOH is fundamentally about public policy directed at reducing structural inequalities, then who is to blame for not facilitating action—public health workers who feel most comfortable in individual, behaviorally-oriented preventive activities; civil society organizations who should be advocating for enlightened policies to the policy elite and/or motivating the public to call for such action; health and health-related decision makers who perpetuate downstream responses; researchers who fail to translate the overwhelming body of research to all of the above; or should we blame a hegemonic sociopolitical system that is shifting ever more to the right? Proponents of the SDOH can easily state what needs to be done: In order to develop policy that will equitably create health, a common understanding and agreement must exist regarding the pathways through which inequity works to create differential health in society [47]. The CSDH asserted that the underlying causes of health inequalities – the social structures and mechanisms that shape and perpetuate differential access to power and resources – must be addressed in order to tackle unjust health outcomes appropriately [1,5]. In order to effectively draw attention to this idea and begin to take decisive action on differential health outcomes at least in Canada, we lack “know how” to operationalize action on the SDOH for the improvement of population health and the reduction of health inequities.

## Conclusions

This study has called attention to some novel dilemmas about the perception of the SDOH and possible reasons for inaction on the root causes of health inequalities. We found that two groups with different affiliations to formal public health could both discuss the SDOH without acknowledging structural inequity in power and resources when discussing actions that might be used to improve health. We also found, as others have, that those working in public health had difficulty moving beyond individual actions that they or their clients could take to improve health. For a group more focused on advocacy than direct service provision for the improvement of population health, the Ottawa Charter framework seemed more easily suited to their recommendations than calling upon action on the SDOHs. Achievement of one of the key Charter strategies, namely healthy public policies, was still relegated to an educational strategy targeting the public, policy makers and politicians.

While there remain varying perceptions of the SDOH in Canada, as illustrated by this and earlier studies, the way forward may well be in providing tangible population health intervention strategies that have been shown to improve population health and reduce health inequities rather than greater effort in further understanding of what has proved to be a difficult concept. These interventions could then be championed by public health and advocates affiliated with public health. Given that public policy support is aligned with public and political support, future research may as well need to examine how best to position action on the SDOH using methods beyond education of key stakeholders.

## Competing interests

The authors declare that they have no competing interests.

## Authors’ contributions

LMcI oversaw the funding, execution, interpretation, and manuscript preparation for the study. RS led manuscript drafting and synthesis of supportive literature. CN assisted with data collection and drafted interpretative results of the two study phases. HB assisted with study design, data collection, interpretation of results, and value for knowledge translation. LMcL assisted with study design, data collection, interpretation of results, and substantive suggestions in manuscript preparation. All authors read and approved the final manuscript.

## References

[B1] World Health Organization (WHO) Closing the gap in a generation: health equity through action on the social determinants of health 2008 Geneva 10.1016/S0140-6736(08)61690-6 18994664

[B2] Goldman N Social inequalities in health: disentangling the underlining mechanisms Ann N Y Acad Sci 2001 954 118 139 11797854

[B3] Pearce N Davey Smith G Is social capital the key to inequalities in health? Am J Public Health 2003 93 122 129 10.2105/AJPH.93.1.122 12511401 PMC1447706

[B4] Peter F Health equity and social justice J Appl Philos 2001 18 159 170 10.1111/1468-5930.00183 11785544

[B5] Chapman AR The social determinants of health, health equity, and human rights Health Hum Rights 2010 12 17 30 21178187

[B6] McIntosh CN Finès P Wilkins R Wolfson MC Income disparities in health-adjusted life expectancy for Canadian adults, 1991 to 2001 Health Rep 2009 20 55 64 20108606

[B7] Adler N Stewart J Cohen S Cullen M Roux AD Dow W Evans G Kawachi I Marmot M Matthews K McEwen B Schwartz J Seeman T Williams D The John D. and Catherine T. MacArthur Foundation Research Network on Socioeconomic Status and Health Reaching for a healthier life: facts on socioeconomic status and health in the US 2007 San Francisco [http://www.macses.ucsf.edu/downloads/reaching_for_a_Healthier_life.pdf]

[B8] Department of Health Tackling health inequalities: a programme for action 2003 http://webarchive.nationalarchives.gov.uk/+/www.dh.gov.uk/en/publicationsandstatistics/publications/publicationspolicyandguidance/dh_4008268

[B9] National Collaborating Centre for Determinants of Health Integrating social determinants of health and health equity into Canadian public health practice: environmental scan 2010 2010 Antigonish, NS

[B10] Public Health Agency of Canada (PHAC) What makes Canadians healthy or unhealthy? 2013 [http://www.phac-aspc.gc.ca/ph-sp/determinants/determinants-eng.php#income]

[B11] Senate Subcommittee on Population Health A healthy productive Canada: a determinant of health approach 2009 Ottawa

[B12] Chief Public Health Officer Chief Public Health Officer’s report on the state of public health in Canada, 2008: addressing health inequalities 2008 [http://www.phac-aspc.gc.ca/publicat/2008/cphorsphc-respcacsp/pdf/CPHO-Report-e.pdf]24027247

[B13] Conference Board of Canada Healthy people, healthy performance, healthy profits: The case for business action on the socio-economic determinants of health 2008 Ottawa http://www.conferenceboard.ca/e-library/abstract.aspx?did=2818

[B14] Raphael D Curry-Stevens A Bryant T Barriers to addressing the social determinants of health: insights from the Canadian experience Health Policy 2008 88 222 235 10.1016/j.healthpol.2008.03.015 18471923

[B15] Raphael D Escaping from the phantom zone: social determinants of health, public health units and public policy in Canada Health Promot Int 2009 24 193 198 10.1093/heapro/dap005 19252200

[B16] Bryant T Raphael D Schrecker T Labonte R Canada: a land of missed opportunities for addressing the social determinants of health Health Policy 2011 101 1 44 58 10.1016/j.healthpol.2010.08.022 20888059

[B17] Collins P Hayes M Twenty years since Ottawa and Epp: researchers' reflections on challenges, gains, and future prospects for reducing health inequities in Canada Health Promot Int 2007 22 4 337 345 10.1093/heapro/dam031 17977903

[B18] Low J Theriault L Health promotion policy in Canada: lessons forgotten, lessons still to be learned Health Promot Int 2008 23 2 200 206 10.1093/heapro/dan002 18227503

[B19] Eyles J Brimacombe M Chaulk P Stoddart G Pranger T Moase O What determines health? To where should we shift resources? Attitudes towards the determinants of health among multiple stakeholder groups in Prince Edward Island, Canada Soc Sci Med 2001 53 1611 1619 10.1016/S0277-9536(00)00445-7 11762887

[B20] Raphael D Barriers to addressing the societal determinants of health: public health units and poverty in Ontario, Canada Health Promot Int 2003 4 397 405 14695371 10.1093/heapro/dag411

[B21] Reutter L Neufeld A Harrison MJ Public perceptions of the relationship between poverty and health Can J Public Health 1999 90 13 18 10189732 10.1007/BF03404091 PMC6979740

[B22] Rock MJ McIntyre L Persaud SA Thomas KL A media advocacy intervention linking health disparities and food insecurity Health Ed Res 2011 26 6 948 960 10.1093/her/cyr043 PMC3219881 21685402

[B23] Lavis JN Ross SE Stoddart GL Hohenadel JM McLeod CB Evans RG Do Canadian civil servants care about the health of populations? Am J Public Health 2003 93 4 658 663 10.2105/AJPH.93.4.658 12660214 PMC1447807

[B24] Kahan BR Goodstadt MS Understanding the determinants of health: key decision makers in Saskatchewan Health districts and Saskatchewan Health, 1998 Can J Public Health 1999 90 S47 S52 10686761 10.1007/BF03403580 PMC6979974

[B25] Collins P Do great local minds think alike? Comparing perceptions of the social determinants of health between non-profit and governmental actors in two Canadian cities Health Educ Res 2012 27 3 371 384 10.1093/her/cys009 22319077

[B26] Hayes M Ross IE Gasher M Gutstein D Dunn JR Hackett RA Telling stories: news media, health literacy and public policy in Canada Soc Sci Med 2007 64 1842 1852 10.1016/j.socscimed.2007.01.015 17337317

[B27] Gasher M Hayes MV Ross I Hackett RA Gutstein D Dunn JR Spreading the news: social determinants of health reportage in Canadian daily newspapers Can J Commun 2007 32 557 574

[B28] Picard A *Universal health care scores well, but don’t be deluded* *The Globe and Mail 2011.* http://www.theglobeandmail.com/life/health-and-fitness/universal-health-care-scores-well-but-dont-be-deluded/article622021/

[B29] Picard A *Social injustice can kill, global panel claims* *The Globe and Mail 2008.* http://www.theglobeandmail.com/life/social-injustice-can-kill-global-panel-claims/article1060222/

[B30] Raphael D The Politics of Population Health: Why the Welfare State Is the Key Social Determinant of Health 2003 Toronto, Canada: York University

[B31] Coburn D Income inequality, social cohesion and the health status of populations: the role of neo-liberalism Soc Sci Med 2000 51 135 146 10.1016/S0277-9536(99)00445-1 10817476

[B32] Lavis J Perspective: ideas at the margin or marginalized ideas? Nonmedical determinants of health in Canada Health Aff 2002 21 107 112 10.1377/hlthaff.21.2.107 11900150

[B33] Lavis J Research, public policymaking, and knowledge-translation processes: Canadian efforts to build bridges J Contin Educ Health Prof 2006 26 37 45 10.1002/chp.49 16557509

[B34] Lomas J The in-between world of knowledge brokering BMJ 2007 26 1 129 132 17235094 10.1136/bmj.39038.593380.AE PMC1779881

[B35] Waddell C Lavis JN Abelson J Lomas J Shepherd CA Bird-Gayson T Giacomini M Dan Offord DR Research use in children’s mental health policy in Canada: maintaining vigilance amid ambiguity Soc Sci Med 2005 61 1649 1657 10.1016/j.socscimed.2005.03.032 16029772

[B36] Sandelowski M Whatever happened to qualitative description? Res Nurs Health 2000 23 4 334 340 10.1002/1098-240X(200008)23:4<334::AID-NUR9>3.0.CO;2-G 10940958

[B37] Palys T Given LM Purposive sampling The Sage Encyclopedia of Qualitative Research Methods. Volume 1 2008 Thousand Oaks, CA: Sage 697 698

[B38] Aronson J A pragmatic view of thematic analysis The Qualitative Report 1994 2 1 [http://www.nova.edu/ssss/QR/BackIssues/QR2-1/aronson.html]

[B39] Lloyd SL Using comprehension strategies as a springboard for student talk J Adolesc Adult Lit 2004 48 114 124 10.1598/JAAL.48.2.3

[B40] McCloskey R A guide to discourse analysis Nurse Res 2008 16 24 43 10.7748/nr2008.10.16.1.24.c6751 19025104

[B41] World Health Organization (WHO) The Ottawa charter for health promotion 1986 Ottawa [http://www.who.int/healthpromotion/conferences/previous/ottawa/en/index.html]

[B42] Raphael D A discourse analysis of the social determinants of health Critical Public Health 2011 21 2 221 236 10.1080/09581596.2010.485606

[B43] Epp J Achieving health for all: a framework for health promotion Health Promot Int 1986 1 419 428 10.1093/heapro/1.4.419 10302169

[B44] Hamilton N Bhatti T Health Promotion Development Division Population health promotion: an integrated model of of population health and health promotion 1996 Ottawa, ON: Public Health Agency of Canada

[B45] Raphael D Getting serious about the social determinants of health: new directions for public health workers Global Health Promot 2008 15 30 15 20 10.1177/1025382308095650 18784048

[B46] Wilson J Wilson D Positivism Social theory 1983 Englewood Cliffs, NJ: Prentice Hall 11 18

[B47] Daniels N Kennedy BP Kawachi I Why justice is good for our health: the social determinants of health inequalities Daedalus 1999 128 215 251 11645876

